# Biomarkers of Adverse Response to Mercury: Histopathology versus Thioredoxin Reductase Activity

**DOI:** 10.1155/2012/359879

**Published:** 2012-07-19

**Authors:** Vasco Branco, Paula Ramos, João Canário, Jun Lu, Arne Holmgren, Cristina Carvalho

**Affiliations:** ^1^Research Institute for Medicines and Pharmaceutical Sciences (iMed.UL), Faculty of Pharmacy, University of Lisbon, Avenue Professor Gama Pinto, 1649-003 Lisbon, Portugal; ^2^Marine Environment and Biodiversity Unit, Institute for Sea and Atmospheric Research (IPIMAR/IPMA), Avenue Brasília, 1440-006 Lisbon, Portugal; ^3^Aquaculture Unit, Institute for Sea and Atmospheric Research (IPIMAR/IPMA), Avenue Brasília, 1440-006 Lisbon, Portugal; ^4^Department of Medical Biochemistry and Biophysics, Karolinska Institutet, 17177 Stockholm, Sweden

## Abstract

Exposure to mercury is normally assessed by measuring its accumulation in hair, blood or urine. Currently, the biomarkers of effect that have been proposed for mercurials, such as coproporphyrines or oxidative stress markers, are not sensitive enough and lack specificity. Selenium and selenoproteins are important targets for mercury and thioredoxin reductase (TrxR) in particular was shown to be very sensitive to mercury compounds both *in vitro* and *in vivo*. In this study we looked into the relation between the inhibition of thioredoxin reductase (TrxR) activity and histopathological changes caused by exposure to mercurials. Juvenile zeabra-seabreams were exposed to Hg^2+^ or MeHg for 28 days and histopathological changes were analyzed in the liver and kidney as well as TrxR activity. Both mercurials caused histopathological changes in liver and kidney, albeit Hg^2+^ caused more extensive and severe lesions. Likewise, both mercurials decreased TrxR activity, being Hg^2+^ a stronger inhibitor. Co-exposure to Hg^2+^ and Se fully prevented TrxR inhibition in the liver and reduced the severity of lesions in the organ. These results show that upon exposure to mercurials, histopathological alterations correlate with the level of TrxR activity and point to the potential use of this enzyme as a biomarker of mercury toxicity.

## 1. Introduction

Adverse health effects of mercury include neurotoxicity, nephrotoxicity, cardiotoxicity, teratogenicity and immunotoxicity. However, the molecular mechanisms underlying mercury toxicity remain unclear with detrimental consequences on the development and validation of appropriate biomarkers of predictive toxic effects. Neurotoxic symptoms are the most visible aspect of mercury poisoning [1]. Nevertheless, the liver and kidney also accumulate high amounts of mercurials [2–4] that may impair their regular functioning. As happens with most xenobiotics, mercury compounds are mainly metabolized in the liver, where demethylathion [5, 6] and conjugation with Se [7, 8] or glutathione can occur [1]. In the liver of animals exposed to high levels of mercurials, hepatocytes are frequently hypertrophied with large-size vacuoles and widespread areas of necrosis can often be observed [3, 9]. The proximal tubule is the kidney structure most affected by mercurials; the cellular changes include swelling of the mitochondrial matrix and endoplasmatic reticulum, loss of membrane integrity and eventual cellular necrosis [10]. Nephrotoxicity caused by to Hg^2+^ accumulation, is well recognized but may also arise from MeHg exposure [10, 11]. 

In risk assessment, biomarkers are important tools to assess the exposure, effect or susceptibility of individuals to a given xenobiotic. In the case of mercury compounds, only the use of biomarkers of exposure, such as the determination of mercury levels in hair [12, 13], blood [14, 15] and urine [16, 17] is generalized. However, the correlation between symptoms and Hg levels in hair, blood or urine is not always evident due to inter-individual variability in susceptibility to mercury [17] and to the delayed onset of effects that characterizes mercury poisoning [1]. Mercury toxic effects are frequently evaluated in humans and animals by conducting psychological and motor tests to assess the degree of neurological damage [17–19] although, changes in mental and motor skills might signify that mercury impairment of biological functions is already established. Therefore, the imposing challenge is still to identify a biomarker predictive of effect for mercury. Coproporphyrines levels and their excretion pattern in urine were proposed to evaluate early effects of mercurials [20, 21], but they have not proven to be a sensitive and useful indicator of mercury toxicity [19] and its use has been quite limited. 

The affinity of mercury to thiol groups (–SH) makes peptides and proteins vulnerable to its presence, especially when sulfhydryl groups are in the active site of enzymes. Changes in the activity of several enzymes involved in antioxidant action, such as glutathione reductase (GR) [22], superoxide dismutase (SOD) [23], and catalase (CAT) [24] are indicative of mercury induced oxidative stress. Glutathione depletion [25, 26], resulting from complexation with mercurials is also a good but unspecific indicator of mercury effects. Metallothionine induction [27] is a biochemical change that has been previously related to mercury exposure. Nevertheless, these changes are not specific and do not allow to distinguish mercury related effects in a multi-contaminant context.

Selenols (–SeH) have a lower pKa than thiols (5.3 versus 8.5) and under physiological conditions are fully ionized to selenolates (–Se^−^) and thus are more reactive and can easily interact with mercury [28]. Selenoenzymes such as glutathione peroxidases (GPxs) are good targets for mercury [29–32] but, recently [28], the involvement of the thioredoxin system-comprising thioredoxin (Trx), the selenoenzyme thioredoxin reductase (TrxR) and NADPH-on the molecular mechanism of mercury toxicity was proven. The inhibitory effects of mercurials on the thioredoxin system have been shown both *in vitro* [28, 33] and *in vivo* [4, 34, 35]. Thioredoxin reductase is particularly sensitive to mercurials which results from its highly nucleophylic structure. Reduced TrxR has two active sites in each homodimer that include a dithiol in the FAD domain and a selenolthiol in the interface domain [34, 36]. By contrast, the homologous enzyme GR, which differs from TrxR by lacking Se in the C-terminal active site, is not inhibited in the presence of mercury compounds [4, 28]. Given the importance of the TrxR and the thioredoxin system to several cellular functions such as protein repair and regulation of the cellular cycle [36], we hypothesize that TrxR inhibition might be a key mechanism by which mercury toxicity develops. Thus, this work investigates the incidence of histopathological changes in the liver and kidney of zeabra-seabreams and its correlation with the decrease in TrxR activity caused by MeHg or Hg^2+^ exposure. The influence of Se co-exposure and post-exposure treatment on enzyme activity and on the alterations observed is also discussed.

## 2. Materials and Methods

### 2.1. *In Vivo* Assays

Zeabra-seabreams (*Diplodus cervinus*), were used as a model. This species is easy to handle in captivity and, since the organs of fishes are similar to those of mammals, they constitute a good alternative to rodents as a model [37]. A total of 63 fishes were divided into 6 experimental groups: control (C; *n* = 12); selenium (Se, provided as sodium selenite; *n* = 9); exposure to Hg^2+^ (HgII; *n* = 12); exposure to MeHg (MeHg; *n* = 12); co-exposure to Hg^2+^ and Se (HgSe; *n* = 12); co-exposure to MeHg and Se (MeHgSe; *n* = 12). Juvenile zeabra-seabreams were kept in tanks at a density of 2.5 g of fish per liter. Oxygen saturation, in water was kept close to 100% and ammonia and pH were kept within normal limits. Exposure concentrations were set at 2 *μ*g L^−1^ for both Hg^2+^ and MeHg and at 10 *μ*g L^−1^ for Se. Exposure lasted 28 days and was followed by 14 days of depuration. During the depuration, fishes from HgII and MeHg groups were divided into two sub-groups, one kept in clean water and the other one kept in water supplemented with Se (HgRSe and MeHgRSe). The experiment was described in detail in Branco et al. [34].

### 2.2. Organ Collection

Sampling of fish took place at days 14, 28 and 42 (hereafter referred as d14, d28 and d42). Three fishes were taken from each group at each sampling day. The liver and kidney were collected and rinsed with 0.9% NaCl. Sub-samples of these organs (were immediately fixed in 10% neutral buffered formalin (diluted in 10% salt water) for histopathological observation and the remaining organs frozen at −80°C until analysis.

### 2.3. Histopathology Analysis

Following fixation in 10% neutral buffered formalin, samples were washed with distilled water and dehydrated in a progressive series of ethanol, embedded in paraffin and then cut into 3 *μ*m thick sections further stained with Harris haematoxylin (Merck) and counterstained with Eosin (Merck) according to the standard method described by Lillie and Fullmer [39]. Histopathological observations were carried out by using a Olympus BX 51light microscope linked to a digital camera (Olympus DP-20).

The Organ Damage Index (ODI) was calculated for each exposure group at d28 and d42 according to the formula:

(1) ODI=∑n=14Pi ×NOL,

where Pi is the pathological importance factor following the proposal of Bernet et al. [38] for a given organ lesion (OL) observed in the number of fishes (*N*) tested at each time-point. 

### 2.4. Total Protein Determination

Organ samples were homogenized with a glass mortar and Teflon pestle in TE buffer (pH 7.5) containing a protease inhibitor cocktail (Roche), followed by centrifugation for 7 min at 12,000 rpm and 4°C. The pellet was discarded and supernatants used for enzyme activity assays. The total amount of protein in samples was determined in the supernatant fraction by measuring absorption at 595 nm in a microplate reader, according to Bradford [40], using Coomassie Brilliant Blue G-250 dye (Bio-Rad). Concentration of protein was quantified by using a calibration curve prepared by sequential dilution of a BSA standard solution.

### 2.5. Thioredoxin Reductase Activity

The activity of TrxR was determined using the insulin reduction endpoint assay proposed by Arnér and Holmgren [41]. Samples were incubated with TE buffer, fully reduced human Trx (3 *μ*M; IMCO Corp. Sweden), insulin (0.3 mM), NADPH (2.5 mM), EDTA (2.5 mM) and HEPES (85 mM; pH 7.6) for 20 min at room temperature. Control wells containing the same mixture but without added Trx were simultaneously prepared. After incubation, the reaction was stopped by adding 250 *μ*L of a 1 mM DTNB solution in 6 M guanidine*·*HCl and absorbance at 412 nm was measured in a microplate reader. 

### 2.6. Glutathione Reductase Activity

For GR activity, supernatants were incubated in 96-well plates with phosphate buffer (100 mM; pH 7.0), NADPH (1 mM), and GSSG (200 *μ*M). The reaction was monitored for 5 min at 30°C in a microplate reader and the decrease in absorption at 340 nm, resulting from NADPH oxidation was registered [42]. 

### 2.7. Statistical Analysis

Differences between groups were assessed by computing the Mann-Whitney test for independent samples. Differences were considered significant at a *P* value below 0.05 [43].

## 3. Results and Discussion

### 3.1. Liver Histopathology

**Figure 1:**
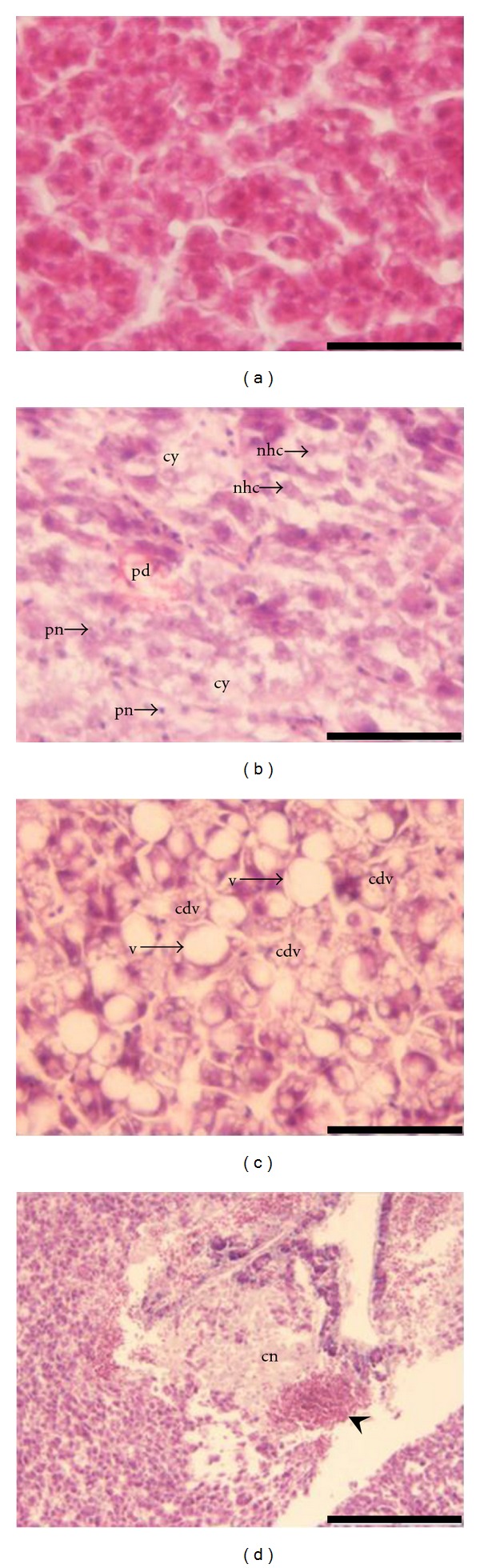
Histopathological observations in the liver of* Diplodus cervinus* after 28 days of exposure. (a) control group: section of polygonal hepatocytes cords (bar = 50 *μ*m); (b) exposure to Hg^2+^: extensive necrosis with congestion of sinusoids and pigment deposition (pd), nuclear hypercromatose (nhc), picnotic nucleus (pn) and cytolysis (cy) (bar = 50 *μ*m); (c) exposure to MeHg: extensive cytoplasmic degenerative vacuolization (cdv) with large, smooth-edged vacuoles (v) (bar = 50 *μ*m); (d) co-exposure to MeHg and Se: focal coagulative necrosis (cn) associated to blood congestion (arrowhead) (bar = 200 *μ*m).

Fish dissection showed that the liver of fishes from MeHg and HgII groups had softer consistency, when compared with the C group. No lesions or alterations were observed in the liver of controls (Figure 1(a)). The liver of fishes exposed for 28 days to MeHg and Hg^2+^ showed signs of hepatocyte alterations, namely, degenerative cytoplasm alterations, architectural pattern changes, loss of typical polygonal cell shape and undefined cell limits. Additionally, vacuolar degeneration with lateral migration of the nuclei, hydropic degeneration (Figure 1(b)), vacuolization within the hepatocytes with lipid-type vacuoles, which can be infiltrated fats, and appearance of some typical globular bodies may result from an increase in the lipid, water and/or glycogen content [3]. Hypertrophied hepatocytes were also disseminated at the parenchyma in fish from both HgII and MeHg groups, which is in good agreement with alterations described in the literature [3, 44, 45]. Liver extensive focal necrosis associated to congestion and pigments deposition was observed in two fishes belonging to the HgII group (Figure 1(c)). The MeHg group displayed three cases of little focal liver necrosis. During exposure to both mercurials the type of lesions was the same, but the necrotic lesions observed in the liver of fishes exposed to Hg^2+^ were more predominant and severe, leading to higher ODI values (Table 1). Despite the fact that, the accumulation of Hg^2+^ was lower than MeHg (Table 2), the ODI was higher. This result contrasts with the observations by Ribeiro et al. [45] where Hg^2+^ failed to cause any significant liver change in the artic charr, in comparison to MeHg. However, it should be stressed that Ribeiro et al. [45], used oral administration of mercurials with food and in those circumstances Hg^2+^, is much less absorbed in the GI tract than MeHg [1]. Although fishes from HgSe and MeHgSe groups (Figure 1(d)) showed at d28 the same kind of lesions observed in fish exposed only to mercurials (i.e, focal cellular vacuolization, megalocytic hepatocytes focal necrosis and congestion of the hepatic parenchyma), these lesions were observed side by side with normal cells.

After the depuration phase at d42, fish exposed to mercurials still exhibited extensive coagulative necrotic changes. Hypertrophy of the hepatocytes was clear in the parenchyma outside necrotic areas. Fishes depurating in water containing Se presented the same type of necrotic lesion although liver parenchyma beyond the necrotic zones appeared normal, resulting in a lower ODI than the index attained by fishes depurating in clean water (Table 1).

### 3.2. Kidney Histopathology

**Figure 2:**
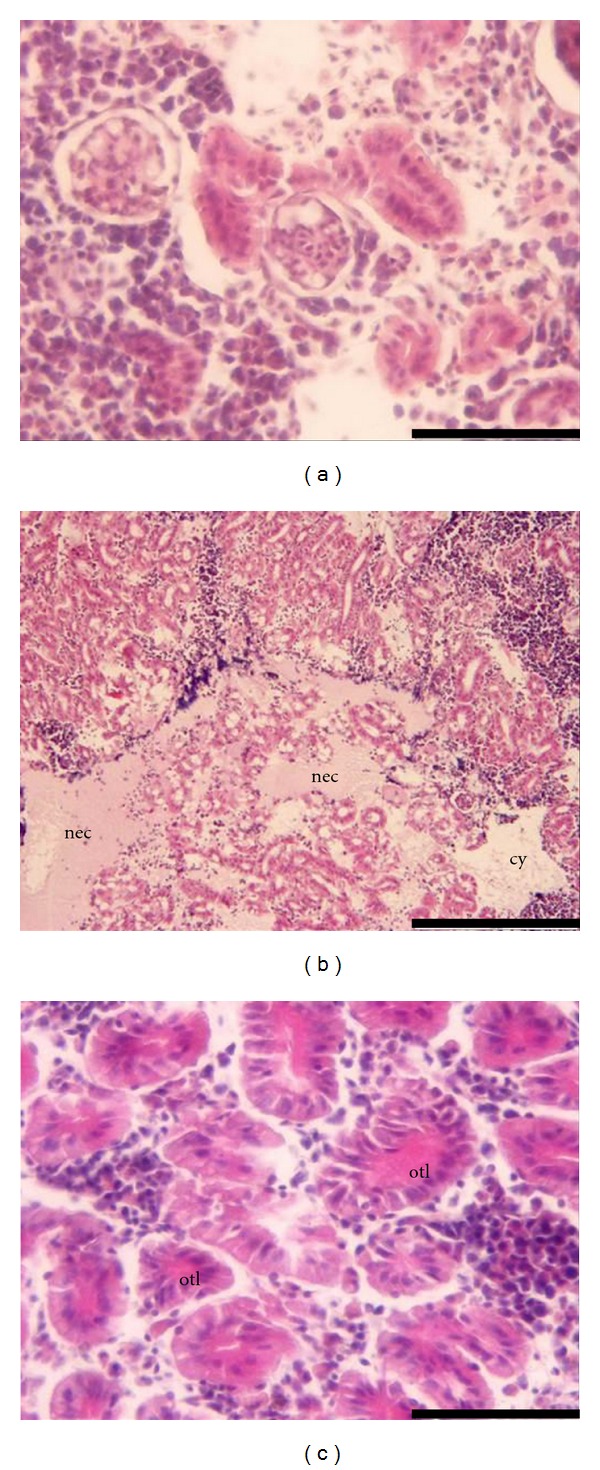
Histopathological observations in the kidney of *Diplodus cervinus* after 28 days of exposure to Hg^2+^. (a) control group: posterior kidney section (bar = 50 *μ*m); (b) exposure to Hg^2+^: posterior kidney section showing a massive necrosis area (nec) and cytolysis (cy) (bar = 200 *μ*m). (c) exposure to Hg^2+^: occlusion of the tubular lumen (otl) (bar = 50 *μ*m).

Kidney changes observed in fish exposed to both mercurials at days 28 and 42 were vacuolar and hydropic degeneration of tubular epithelium and pigment deposits around the tubules. Comparatively, posterior kidney is quite more susceptible to Hg^2+^ which was evidenced by larger necrosis areas (Figure 2(b)) and occlusion of the tubular lumen with eosinophilic material (Figure 2(c)). Co-exposure to MeHg and Se seemed to delay the appearance of the more severe lesions in the kidney (Table 1). Co-exposure to Hg^2+^ and Se decreased the detrimental effects of mercury at the end of the depuration period (Table 1). This decrease in renal toxicity does not reflect a decrease in mercury concentration (Table 2) but instead it might be related to the formation of inorganic inert complexes.

### 3.3. Enzymatic Activities

**Figure 3:**
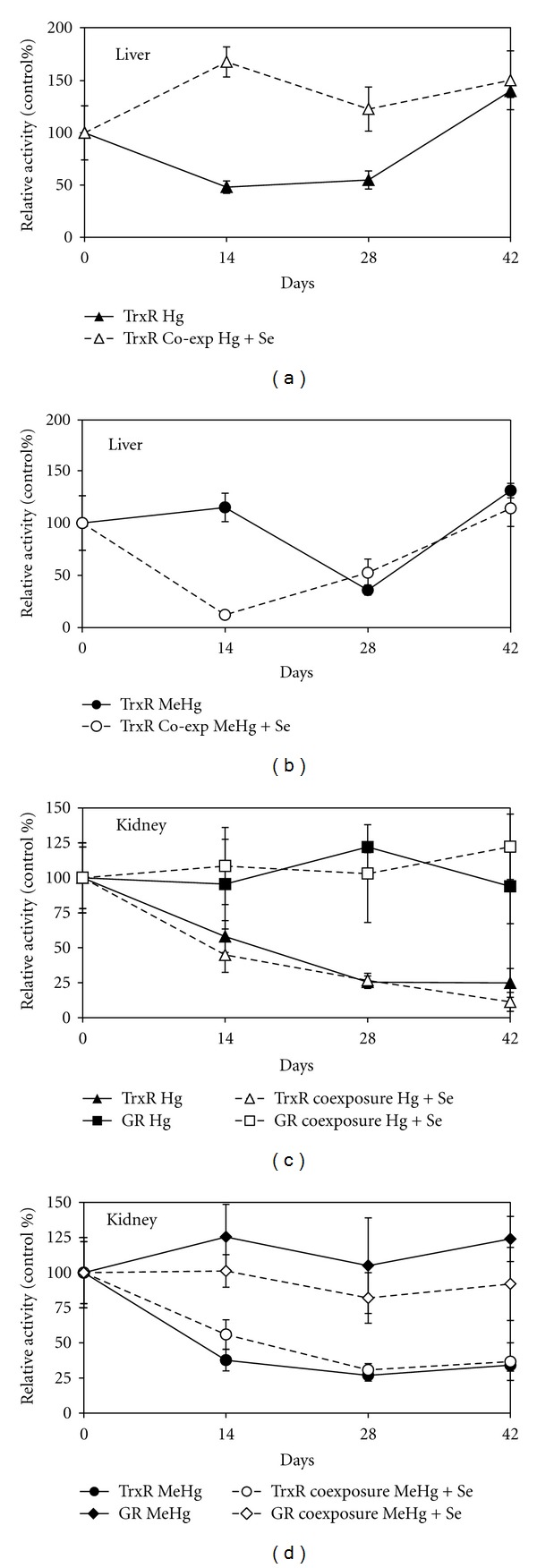
Enzymatic activities in liver and kidney of zeabra-seabreams exposed to mercurials and co-exposed to mercurials and Se (subset of results from a study previously reported in Branco et al. [34]). Exposure lasted 28 days and was followed by 14 days of depuration in clean or Se-supplemented water. (a) TrxR activity in the liver of seabreams exposed to Hg^2+^ and co-exposed to Hg^2+^ and Se; (b) TrxR activity in the liver of seabreams exposed to MeHg and co-exposed to MeHg and Se; (c) TrxR and GR activities in the kidney of seabreams exposed to Hg^2+^ and co-exposed to Hg^2+^ and Se; (d) TrxR and GR activities in the kidney of seabreams exposed to MeHg and co-exposed to MeHg and Se. GR activity in the liver did not show significant variation and was not represented to improve clarity.

The activity of TrxR in the liver and kidney of zeabra-seabreams is shown in Figure 3. In the liver, TrxR activity was decreased by 52% at d14 (*P* < 0.05; Figure 3(a)) during Hg^2+^ exposure, while in the case of MeHg, the inhibition was only observed by d28 (*P* < 0.05; Figure 3(b)). As previously shown and discussed [28, 34, 36] Hg^2+^ is a stronger inhibitor of TrxR than MeHg. Recovery of TrxR activity was complete at the end of depuration and was not influenced by Se supplementation during this period (data not shown). Co-exposure to Se besides reducing the ODI index, clearly prevented the inhibitory effect of Hg^2+^ over TrxR activity, that is, activity levels do not differ significantly from the control (*P* > 0.05; Figure 3(a)), but was not effective over MeHg inhibition (Figure 3(b)) whose toxic effects seem to be increased by Se. *In vitro* experiments also confirmed that the co-exposure to MeHg and Se increased the detrimental effects over the TrxR activity [34]. At the same time it was shown that Se can remove Hg^2+^ bound to the selenolthiol in the active site of TrxR restoring activity while MeHg showed no displacement from the active site [34]. Further studies to fully elucidate the contradictory role of Se in the presence of different mercury species are being conducted. 

In the kidney, TrxR activity was significantly affected (*P* < 0.05) both in HgII (42% inhibition) and MeHg (62% inhibition) groups at d14 and the inhibitory effect remained throughout the entire experiment (Figures 3(c) and 3(d)). Selenium showed no protective effect during co-exposure or on the recovery of TrxR activity during the depuration phase. As we have previously suggested [36], the protective Se over TrxR is organ specific and might be related to both the Se : Hg ratio and the capacity of selenoenzyme expression within the organ. In the liver, besides higher Se : Hg ratios [36] the level of selenoenzymes expression is assumed to be higher [44]. It should be stressed that, albeit TrxR activity did not recover, the citotoxic effects of MeHg decreased with Se co-administration in part due to the lower MeHg accumulation in both organs, liver and kidney, which reflected in the ODI values (Table 2). Since TrxR presents a high affinity for mercurials, any available mercury will primarily bound the selenolthiol of its active site and therefore it is normal that the inhibition of TrxR is observed in cases where cito-/organ toxicity is largely decreased (Table 1).

For the HgII group possible explanations for the decreased renal toxicity in the presence of Se include the formation of inorganic inert complexes and the participation of Se in antioxidant in cellular pathways. GR activity was not inhibited in the liver (results not shown) or in the kidney (*P* > 0.05; Figures 3(c) and 3(d)) in any group. On the contrary, the slight increase of GR activity seems to be a compensation mechanism for the loss of antioxidant protection provided by TrxR. Moreover, the fact that GR is homologous to TrxR, but lacks the Sec residue in the active site, reinforces the importance of TrxR active site as a main target for mercurials. 

## 4. Conclusions

Mercury effects have been evaluated using different types of biomarkers. However, these are normally non-specific or are not predictive of toxicity but, instead, correspond to manifestations of toxicity itself. The thioredoxin system is responsible for several key cellular functions that range from anti-oxidant defense to regulation of the cellular cycle [33] and loss of activity will result in apoptosis [46]. Mercury (II) was shown to be a stronger inhibitor of TrxR than MeHg and also produced the most extensive array of organ lesions (Table 1). Also, when zeabra-seabreams were co-exposed to Se and Hg^2+^ the severity of the lesions in the liver decreased while TrxR activity was kept at normal levels. In the cases of exposure to Hg^2+^ and MeHg, although we observed full recovery of the activity of TrxR in the liver following depuration, the histopathological lesions reversion was not significant. Possibly, cellular mechanisms downstream of the thioredoxin system were affected to such a degree that recovery of TrxR activity does not reflect in an immediate organ recovery and time will be needed to replace damaged cellular structures. Overall, this work shows that the strong inhibition of TrxR activity is related with the histopathological alterations displayed in the liver and kidney of seabreams indicating the potential use of TrxR as a biomarker of effect of mercury toxicity.

## Figures and Tables

**Table 1:** Values of the organ damage index^∗^ (ODI) calculated for the liver and kidney of zeabra-seabreams exposed to the different treatments. No lesions were observed in control fishes. MeHg: exposure to Methylmercury; HgII: exposure to Hg^2+^; MeHgSe: coexposure to MeHg and Se; HgSe: coexposure to Hg^2+^ and Se; MeHgRSe: exposure to MeHg followed by exposure to Se during depuration; HgRSe: exposure to Hg^2+^ followed by exposure to Se during depuration; Se: exposure to selenium.

	Organ lesion (OL)	Pi	MeHg		HgII		MeHgSe		HgSe		MeHgRSe	HgRSe	Se	
			d28	d42	d28	d42	d28	d42	d28	d42	d42	d42	d28	d42
			*N*_OL_	*N*_OL_	*N*_OL_	*N*_OL_	*N*_OL_	*N*_OL_	*N*_OL_	*N*_OL_	*N*_OL_	*N*_OL_	*N*_OL_	*N*_OL_
Liver	Architectural pattern lost	1	4	3	4	4	3	2	4	0	3	3	2	0
	Vacuolar degeneration	1	3	1	4	1	2	0	1	2	3	3	2	0
	Hydropic degeneration	1	4	2	3	1	1	2	1	1	0	3	0	0
	Hypertrophy of hepatocytes	2	2	3	1	4	4	0	4	1	0	0	0	0
	Extensive focal necrosis	3	0	3	2	4	0	2	1	3	0	2	0	0
	Focal necrosis	2	3	0	0	0	1	2	2	0	3	1	0	0
	Parenchymal congestion	1	0	1	2	0	1	0	3	0	0	0	0	0
	Pigment deposition	1	0	0	2	0	0	0	0	0	0	0	0	0

	ODI		21	22	23	26	17	14	24	14	12	17	4	0

Kidney	Vacuolar degeneration	1	1	0	1	2	1	1	0	1	1	2	0	0
	Hydropic degeneration	1	3	2	1	0	1	3	0	3	0	2	0	0
	Pigment deposition	1	3	1	1	1	0	2	0	1	1	0	0	0
	Parenchymal necrosis	3	0	0	0	3	0	0	1	0	0	0	0	0

	ODI		7	3	3	12	2	6	3	5	2	4	0	0

Pi: Pathological index of each lesion according to their importance to organ function [38].

*N*_OL_: number of fishes displaying one type of organ lesion (OL).

^∗^ODI: sum of lesions observed for each organ taking into account their relative severity and the number of fishes (*N*_OL_).

**Table 2:** Quantification of total mercury (HgT) and selenium (Se) values (*μ*g g^−1^) in liver and kidney of zeabra-seabreams exposed to different treatments at days 28 and 42. Results were previously reported in Branco et al. [34]. MeHg: exposure to Methylmercury; HgII: exposure to Hg^2+^; MeHgSe: co-exposure to MeHg and Se; HgSe: co-exposure to Hg^2+^ and Se; MeHgRSe: exposure to MeHg followed by exposure to Se during depuration; HgRSe: exposure to Hg^2+^ followed by exposure to Se during depuration; Se: exposure to selenium; C: control group.

		MeHg		HgII		MeHgSe		HgSe		MeHgRSe	HgRSe	Se		C	
		d28	d42	d28	d42	d28	d42	d28	d42	d42	d42	d28	d42	d28	d42
Liver	HgT (*μ*g g^−1^)	10.2 ± 4.8	6.2 ± 1.4	1.4 ± 0.2	2.8 ± 0.4	5.8 ± 1.7	4.8 ± 1.3	1.5 ± 0.1	2.3 ± 0.5	5.8 ± 1.5	2.4 ± 0.5	0.07 ± 0.02	0.09 ± 0.02	0.10 ± 0.06	0.07 ± 0.03
	Se (*μ*g g^−1^)	0.6 ± 0.1	0.9 ± 0.2	0.5 ± 0.2	1.3 ± 0.2	0.9 ± 0.02	1.3 ± 0.3	1.1 ± 0.3	1.6 ± 0.3	1.3 ± 0.2	1.0 ± 0.1	1.3 ± 0.2	1.3 ± 0.2	1.0 ± 0.14	1.0 ± 0.21

Kidney	HgT (*μ*g g^−1^)	28.7 ± 8.3	17.3 ± 6.3	5.8 ± 1.9	8.3 ± 4.1	15.1 ± 1.9	11.9 ± 3.9	8.6 ± 3.3	10.0 ± 3.5	23.6 ± 1.1	8.8 ± 1.7	0.11 ± 0.03	0.15 ± 0.02	0.16 ± 0.04	0.14 ± 0.01
	Se (*μ*g g^−1^)	1.6 ± 0.6	3.3 ± 0.6	0.4 ± 0.3	1.3 ± 0.2	2.7 ± 1.2	2.1 ± 1.1	1.0 ± 0.3	2.8 ± 0.8	2.3 ± 0.6	4.5 ± 1.2	1.7 ± 0.3	1.7 ± 0.5	1.3 ± 0.6	1.0 ± 0.2
